# Hospital admissions associated with dehydration in childhood kidney transplantation

**DOI:** 10.1007/s00467-023-06095-6

**Published:** 2023-08-09

**Authors:** Amelia K. Le Page, Lilian M. Johnstone, Joshua Y. Kausman

**Affiliations:** 1 Department of Nephrology, Monash Children’s Hospital, Clayton, VIC Australia; 2 Department of Pediatrics, School of Clinical Sciences, Faculty of Medicine, Nursing and Health Sciences, Monash University, Melbourne, Australia; 3 Department of Nephrology, Royal Children’s Hospital, Melbourne, Australia; 4 Murdoch Children’s Research Institute, Royal Children’s Hospital, Melbourne, Australia; 5 Department of Paediatrics, University of Melbourne, Melbourne, Australia

**Keywords:** Dehydration, Kidney transplantation, Children, Hydration

## Abstract

**Background:**

Paediatric kidney transplant recipients may be at a particular risk of dehydration due to poor kidney concentrating capacity and illness associated with poor fluid intake or losses. In this population, creatinine rise may be more likely with relatively mild dehydration, which may trigger hospital admission. This study describes hospital admissions in the first 12 months after transplantation with diagnosis of graft dysfunction associated with dehydration due to illness or poor fluid intake. We assess risk factors for these admissions.

**Methods:**

Data was extracted from medical records of patients transplanted in two tertiary children hospitals. Following descriptive analysis, multiple failure regression analyses were used to identify factors associated with admission for acute kidney allograft dysfunction associated with dehydration.

**Results:**

Of 92 children, 42% had at least 1 dehydration admission in the 12 months following transplantation. Almost half of the dehydration admissions were due to poor fluid intake, which accounted for 1/5 of all unplanned hospital admissions. Target fluid intake at first discharge of > 100 ml/kg/day was associated with dehydration admissions of all types (hazard ratio (HR) 2.04 (95% CI 1.13–3.68)). Teen age was associated with poor fluid intake dehydration admissions (HR 4.87 (95% CI 1.19–19.86)), which were more frequent in mid-summer. Use of enteric feeding tube, which correlated with age under 4, associated with contributing illness dehydration admissions (HR 2.18 (95% CI 1.08–4.41)).

**Conclusions:**

Dehydration admissions in the 12 months following childhood kidney transplantation are common. Highlighted admission risk factors should prompt further study into optimal fluid intake prescription and hydration advice given to children, teenagers, and their carers following kidney transplantation. Use of an enteric feeding tube may not protect patients from admission with dehydration associated with contributing illness.

**Graphical abstract:**

**Figure:**
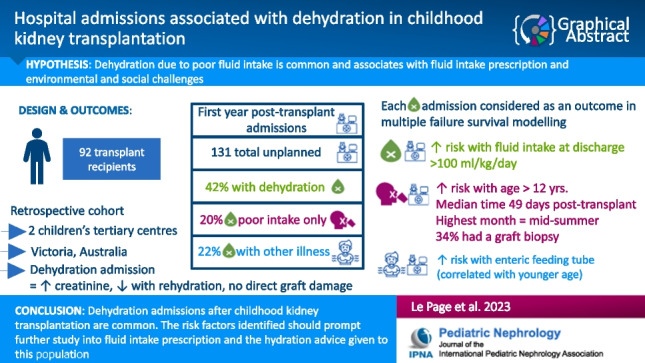
A highger resolution version of the Graphical abstract is available as Supplementary information

**Supplementary Information:**

The online version contains supplementary material available at 10.1007/s00467-023-06095-6.

## Introduction

The paediatric kidney transplant population is at risk of dehydration from acute illnesses such as infections that increase gastrointestinal or other insensible losses [1]. They are also inherently vulnerable to dehydration due to impaired kidney concentrating capacity. This may be related to the transplant kidney [2] or to polyuria from native kidneys [3]. Thus, even in the absence of increased fluid losses, anything that can impact fluid intake may increase the risk of dehydration.

With the single kidney state of most transplants, even mild dehydration may lead to creatinine rise and acute kidney injury (AKI). Early post-transplant, this may lead to rehospitalisation to optimise hydration state, to treat any associated illness, and to exclude acute rejection. It has been our anecdotal experience that such admissions are frequent and that the dehydration is often associated with poor fluid intake.

Despite our anecdotal experience, published evidence of poor fluid intake in children with kidney transplants is very limited. In the non-interventional arm of a trial of an interactive water bottle, fluid intake that was below the target goal was self-reported by more than 60% of 7–19 year-old at a median of 5.83 years post kidney transplant [4]. There is no published data on how often suboptimal fluid intake can lead to dehydration-associated creatinine rise or AKI. There are also no specific reports of frequency of dehydration related to intercurrent illness and AKI in children with kidney transplants. In addition, there are no studies of dehydration-associated hospital admissions. In an American single-centre cohort, readmission with any diagnosis in the first year following paediatric kidney transplant occurred in 79% of patients [5]. These authors categorised primary admission diagnosis as either rejection-related or infection-related, with the latter more common. It is possible that dehydration was a factor in many of these admissions, but this was not specially evaluated in the report.

Readmissions following transplant are an important quality indicator of transplant programmes [6]. In addition, hospitalisation and life participation are identified as important health outcomes for children and their carers [7].

Understanding the frequency and the risk factors for dehydration-associated hospital admissions will identify the extent of the problem and may identify opportunities to prevent these admissions and improve quality of life.

This study was therefore designed to describe hospital admissions with a final diagnosis of graft dysfunction associated with dehydration, due to illness or due to poor fluid intake in the first 12 months after transplantation. We further sought to assess risk factors for these admissions, with particular focus on admissions with dehydration associated with poor fluid intake.

## Methods

### Study design and population

The retrospective cohort included all kidney transplant recipients across two tertiary children hospitals in the state of Victoria in Australia, between January 2011 and December 2021, with a minimum follow-up requirement of 12 months. The study was approved by each hospital’s human research ethics committee as a quality assurance project with requirement for informed consent waived. Medical records were evaluated to identify relevant categories of admission and relevant inpatient and outpatient details to create a fully anonymised dataset. Children with primary graft non-function and multi-organ transplants and those who moved to a transplant centre in another state in the 12-month post-transplant period were excluded.

### Routine transplant immunosuppression and post-operative fluid management

Immunosuppression strategies in both tertiary hospitals in routine low sensitisation risk scenarios involve methylprednisolone and basiliximab as induction, and maintenance triple immunosuppression with mycophenolate, tacrolimus, and prednisolone. Steroid-free regimens are not routinely used but may be considered in low immunologic risk patients at the discretion of the nephrology team. Management of HLA-sensitised children or those with transplant ABO incompatibility typically involves addition of plasma exchange and low-dose IVIG therapies. Some of these children may also receive rituximab. Protocol biopsies are not routinely performed in either institution.

In both tertiary hospitals, fluid management strategies post-operatively are usually based on ml per ml urine output replacement plus insensible losses. Maintaining graft perfusion during surgery after aortic clamp release and in the first 24 h after transplant is a priority, and thus, large volumes of intravenous fluid are typically initially used. There is no fixed protocol for changing fluid intake prescriptions beyond the first 24 h post-transplant, although typically a fixed fluid rate is prescribed which is gradually reduced over successive days whilst watching clinical markers of hydration state, such as weight, and comparing this to pre-operative weight. In the absence of any clinical evidence of fluid overload or dehydration, if weight falls close to pre-operative weight or fluid balance becomes negative, then the fluid target may have been reached. If a minor creatinine rise is seen in conjunction with this fluid weaning prior discharge, this would also be used to indicate that the appropriate fluid target had been reached. Fluid targets provided at discharge may continue to be revised at subsequent appointments according to clinical assessment of hydration state and graft function.

### Outcome

The principal outcome of interest was hospital admission for acute kidney allograft dysfunction associated with dehydration in the first 12 months post-transplant. This was defined as an admission with graft dysfunction where creatinine improved or returned to baseline with rehydration and no other causes for graft dysfunction were identified. Graft dysfunction was defined as an increase (with no specific threshold) in creatinine from the prior three creatinine tests when the patient was well. Admissions with any illness associated with direct graft dysfunction including biopsy-proven acute rejection, BK virus nephropathy (BKVN), pyelonephritis, and obstructive uropathy or admissions associated with calcineurin inhibitor toxicity (defined as a level 30% higher than the upper limit targeted for the specific period post-transplant) were excluded. Two categories of dehydration admission were considered. The first category was where a contributing factor to the dehydration could be identified. This included illnesses with increased fluid losses such as vomiting or diarrhoea, or illnesses with poor fluid intake and/or potential high insensible losses such as respiratory infections. The second category of admission was where no contributing illness could be identified, with poor fluid intake–associated dehydration responsible for the admission. Admissions with creatinine rise during fasting for procedures were excluded. The two admission categories are labelled as *contributing illness dehydration* and *poor intake dehydration*.

Dehydration admission descriptive data of interest included the frequency, timing, and season of admissions post-transplant, and contributing illness diagnosis. Duration of admission and investigations undertaken during the admission including kidney biopsy, ultrasound, and HLA antibody testing were recorded. The percentage creatinine increase at poor intake admission was considered the change from the average of the prior three creatinine tests when the patient was well. AKI was defined and staged according to the 2012 Kidney Disease: Improving Global Outcomes (KDIGO) Clinical Practice Guideline [8]. Frequency of dehydration admissions was presented as a proportion of first year total hospital unplanned admissions for any reason (excluding any planned admissions or day admissions for biopsies, or therapies such as plasma exchange associated with rejection or in association with ABO incompatibility/HLA sensitisation). Admissions following transplant for nephrectomy for the indication of polyuria were reviewed.

### Variables of interest for dehydration admission risk factor analysis

Variables of interest evaluated as potential risk factors for dehydration admissions were chosen based on our anecdotal experience, and a hypothesised risk of exaggerated kidney concentrating defect, poor fluid intake, or risk of fluid loss from illness.

Recipient variables evaluated included the baseline factors gender and age (categorised as < 4 years, 4–12 years, and > 12 years). Age groups were categorised based on the hypothesised risk of dehydration associated with poor fluid intake relating to developmental stage, parental capacity to supervise intake, and pubertal independence. Primary kidney disease was evaluated in categories (congenital anomalies of the kidney and urinary tract (CAKUT), ischaemic kidney disease, nephronophthisis, cystic diseases glomerulonephritis and steroid-resistant and congenital nephrotic syndrome, and others). These categories were chosen based on the hypothesised risk relating to volume of native urine output for some of these diseases, as the specific aetiology of chronic kidney disease (CKD) in native kidneys can be associated with reduced kidney concentrating capacity [9]. We hypothesised that native urine output at each extreme could impact the risk of dehydration. Higher output may be a risk of impaired kidney concentrating capacity post-transplant. Our experience also suggested that at the other extreme of anuria, patients have difficulty adjusting to higher fluid intake post-transplant. We analysed this factor as no anuria or anuria, which was defined as a urine output of nil, under 100 ml per day in teens, or < 0.1 ml/kg/h. Where an accurate 24-h volume was recorded, native urine output was also presented as a continuous variable in volume per weight per hour.

Evaluated transplant factors (at the time of first discharge after transplantation) hypothesised to have a potential impact on renal concentrating defect based on studies of aquaporins in ischaemia reperfusion injury [10] included live versus deceased transplant, and delayed graft function (defined as requiring dialysis within 7 days of transplant). Transplant centre was evaluated to exclude any confounding relating to medical care; however, due to potential privacy implications, specific numerical data is not reported in this paper. Receipt of prior dialysis versus pre-emptive transplantation was hypothesised as a potential risk for poor fluid intake, and the presence of an enteric feeding tube as a potential protective factor. Transplantation during the Australian summer months was evaluated due to potential climate impact on insensible losses in the early phase post-transplant. Use of angiotensin-converting enzyme (ACE) inhibitors at any time in the first 12 months post-transplant was presented as a variable of interest, given potential to exacerbate creatinine rise in the setting of dehydration [11].

We hypothesised that higher discharge target fluid intake would be a risk for dehydration admissions of both types, given that this is a potential marker of reduced renal concentrating capacity, and that a higher target may be difficult for some patients to achieve following discharge. Target fluid intake was categorised as ≤ 100 ml/kg/day or > 100 ml/kg/day. This was chosen based on the median day 3 post-transplant fluid input volumes reported in a British transplant cohort by Wyatt et al. [12].

We hypothesised that early hyperfiltration may be a marker of use of high fluid volumes post-transplant, and potentially also a marker of subclinical fluid overload with relative dilution of creatinine. Hyperfiltration at discharge could therefore indicate a potential risk of creatinine rise with subsequent poor fluid intake. We defined hyperfiltration at discharge as an estimated glomerular filtration rate (eGFR) of 135 ml/min/1.73 m^2^ or more [13], and this was considered a categorical variable. The eGFR at discharge was calculated according to age using the Schwartz or CKD-EPI equations [14, 15].

### Statistical analysis

#### Descriptive analysis

The description of transplant recipients in this cohort was undertaken for each transplant they received and was stratified according to whether a dehydration admission had occurred, the type of dehydration admission (poor intake or contributing illness), and whether there were multiple dehydration admissions. Recipients who received 2 transplants in this study period are thus included twice, in order to describe the cohort at each transplant and evaluate the risk factors unique to each transplant. Where different types of dehydration admissions had occurred in the first 12 months post-transplant, these were included in each of the dehydration admission categories.

Categorical variables are presented as absolute numbers and percentages. Continuous variables are presented as mean (standard deviation (SD)) where normally distributed and median (interquartile range (IQR)) for skewed distribution. Where relevant, population differences (including for missing data) according to admissions were assessed using the Kruskal–Wallis test for continuous data with non-normal distribution and Fisher’s exact test for categorical data.

### Risk factors for dehydration admissions

Each dehydration admission in the 12 months post-transplant was included as an outcome in this analysis, thus enabling the assessment of risk factors for all admissions. The Prentice, Williams and Peterson Total Time (PWPTT) modelling was used, which is a multiple failure outcome variant of the Cox proportional hazard analysis [16]. This modelling method takes into account the potential lack of independence of multiple failure risks within an individual subject by stratifying according to the occurrence of past failures. The model was implemented as presented by Westbury et al. [16].

Time at risk commenced the day after first discharge post-transplant and thereafter was considered for all non-hospital admitted time for the 12-month period after transplantation.

The association of baseline demographic, clinical, or transplant variables of interest with dehydration admissions was evaluated initially in PWPTT univariate models with proportionality assessed using Schoenfeld residuals. Variables with missing data and time-dependent factors were not analysed in these models. Variables were entered into the multivariate model where univariate *p* value was ≤ 0.10, provided that the overall model maintained significance as assessed by the Wald test. Collinearity was assessed using a correlation of coefficients matrix. Variables with a *p* value of < 0.05 in the multivariate models were considered statistically significant.

STATA version 14.2 was used for statistical analyses.

## Results

**Fig. 1:**
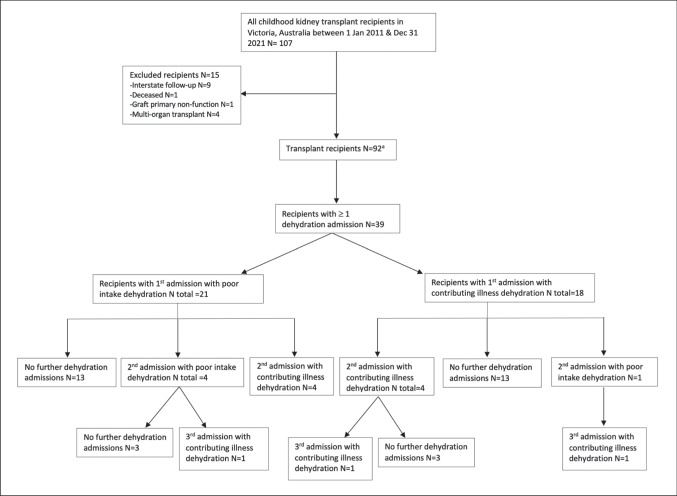
Study cohort dehydration admissions. ^a^One transplant recipient is counted twice here due to having had 2 transplant episodes in the study time frame. N, number of transplant recipients or admissions as indicated

There were 106 children who underwent 107 kidney transplants in Victoria in the study time period. There were 10 children who were excluded due to transfer of medical care to another Australian state within the 12 months post-transplant (Fig. 1).

The final cohort for analysis (Fig. 1) included 92 transplant recipients (one patient had two transplants in the study period).

### Cohort description

**Fig. 2:**
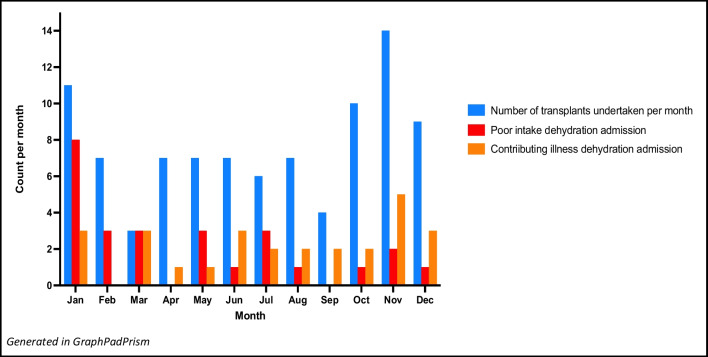
Number of transplants and dehydration admissions over the calendar year

Baseline demographic and transplant characteristics according to the type of dehydration admissions are presented in Table 1. The only missing data was for native urine output volume with accurate data not available for 15/92 recipients. This missing data did not associate with any other analysed variable. There were 16 patients with anuria, with 10 of these having had bilateral native nephrectomies. A slight majority of transplants were undertaken in the spring and summer months (September to March) (55/92 (59.78%)) (Fig. 2). There were 5 patients who had native nephrectomies undertaken post-transplant for the indication of polyuria. Of these, 3 patients had CAKUT as primary kidney disease, 1 patient had cystinosis, and one patient had primary ischaemic kidney disease. For 4 of these patients, their nephrectomies were arranged following a dehydration admission (3/4 were for a poor intake dehydration admission), with 1 patient having the nephrectomy during the original transplant admission. There were no subsequent dehydration admissions for these 5 patients.

**Table 1:** Overall cohort description and according to dehydration admissions

	Total transplant recipients *N* = 92 (91 patients had 92 transplant episodes)	Recipients with no dehydration admissions *N* = 53	Recipients with ≥ 1 dehydration admission of any type *N* = 39	Recipients with ≥ 1 poor intake dehydration admission *N* = 22	Recipients with ≥ 1 contributing illness dehydration admission *N* = 23	Recipients with ≥ 2 dehydration admissions (of any type) *N* = 13
Baseline demographic and clinical factors						
Gender, *N* (%)						
Male	62 (67.39)	32 (60.38)	30 (76.92)	18 (81.82)	16 (69.57)	9 (69.23)
Female	30 (32.61)	21 (39.62)	9 (23.08)	4 (18.18)	7 (30.43)	4 (30.77)
Primary kidney disease, *N* (%)						
CAKUT	45 (48.91)	22 (41.51)	23 (58.97)	14 (63.64)	13 (56.52)	9 (69.23)
Cystic	8 (8.70)	7 (13.21)	1 (2.56)	1 (4.55)	0	0
Nephronophthisis	6 (6.52)	5 (9.43)	1 (2.56)	0	1 (4.35)	0
Glomerulonephritis	4 (4.35)	1 (1.89)	3 (7.69)	1 (4.55)	2 (8.70)	0 (0)
Steroid resistant or congenital nephrotic	10 (10.87)	5 (9.43)	5 (12.82)	2 (9.09)	4 (17.39)	3 (23.08)
Ischaemic	8 (8.70)	4 (7.55)	4 (10.26)	3 (13.64)	2 (8.7)	1 (7.69)
Other^a^	11 (11.95)	9 (16.98)	2 (5.13)	1 (4.55)	1 (4.35)	0 (0)
Age (years, median)	13	12	14	14	8	8
Age category at transplant, *N* (%)						
< 4 years	18 (19.57)	10 (18.87)	8 (20.51)	4 (18.18)	7 (30.43)	5 (38.46)
4–12 years	25 (27.17)	18 (33.96)	7 (17.95)	2 (9.09)	6 (26.09)	2 (15.38)
> 12 years	49 (53.26)	25 (47.17)	24 (61.54)	16 (72.73)	10 (43.48)	6 (46.16)
Native urine output (ml/kg/h, median (IQR))^b^	1.25 (0.32–2.03)	1.11 (0.34–1.81)	1.52 (0.23–2.19)	1.99 (0.66–2.65)	1.12 (0.14–2.04)	1.12 (0.23–1.88)
Anuria^c^ pre-transplant, *N* (%)	16 (17.39)	9 (16.98)	7 (17.95)	4 (18.18)	4 (17.39)	3 (23.08)
Transplant factors						
Past failed transplant, *N* (%)	11 (11.96)	7 (13.21)	4 (10.26)	3 (13.64)	1 (4.35)	1 (7.69)
Deceased donor, *N* (%)	33 (35.87)	23 (43.40)	10 (25.64)	5 (22.73)	6 (26.09)	4 (30.77)
Live donor	59 (64.13)	39 (56.60)	29 (74.36)	17 (77.27)	17 (73.91)	9 (69.23)
No prior dialysis (pre-emptive live donor transplant), *N* (%)	17 (18.48)	11 (20.75)	6 (15.38)	3 (13.64)	3 (13.04)	1 (7.69)
Transplantation during Australian summer, *N* (%)	25 (27.17)	12 (22.64)	13 (33.33)	8 (36.36)	7 (30.43)	3 (23.08)
Gastrostomy or feeding tube at 1st discharge, *N* (%)	31 (33.70)	18 (33.96)	13 (33.33)	5 (22.73)	11 (47.83)	6 (46.15)
ABO or HLA sensitisation, *N* (%)	7 (7.61)	6 (11.32)	1 (2.56)	1 (4.55)	0 (0)	0 (0)
Delayed graft function	15 (16.67)	8 (15.09)	7 (17.95)	2 (10.53)	5 (21.74)	2 (15.38)
Target fluids at discharge, ml/kg (median (IQR))	82.95 (61.52, 107.34)	81.98 (65.57, 103.69)	93.11 (58.14, 114.04)	97.41 (62.5, 114.04)	94.89 (58.14, 111.11)	106.57 (94.89, 114.03)
Discharge target fluid category, *N* (%)						
> 100 ml/kg/day	31 (33.70)	15 (28.30)	16 (41.03)	10 (45.45)	11 (47.83)	8 (61.54)
≤ 100 ml/kg/day	61 (66.30)	38 (71.70)	23 (58.97)	12 (54.55)	12 (52.17)	5 (38.46)
eGFR at discharge, (median (IQR))	84.5 (56.5–126.5)	85 (64–126)	74 (48–127)	87 (59–129)	74 (36–133)	87 (65–129)
Hyperfiltration at discharge, *N* (%) (> 135 ml/min/1.73 m^2^)	16 (17.39)	9 (16.98)	7 (17.95)	4 (18.18)	5 (21.74)	2 (16.67)
Use of ACEI in the study period, *N* (%)	21 (22.83)	11 (20.75)	10 (25.64)	5 (22.73)	6 (26.09)	2 (15.38)

^a^Other primary kidney diseases included cystinosis (2 transplant episodes), aHUS (2 transplant episodes), sepsis-associated CKD (2 episodes), diarrhoea-associated HUS (1 episode), chronic tubulointerstitial diseases (2 episodes), bilateral Wilms-associated nephrectomies (1 episode), and Alport syndrome (1 episode)

^b^Missing data for native urine output in 15/92 recipients

^c^Defined as no native urine output or urine output < 100 ml per day or < 0.1 ml/kg/h

*N*, number of recipients; *CAKUT*, congenital abnormalities of the kidney and urinary tract; *IQR*, interquartile range; *ABO*, ABO blood group; *HLA*, human leukocyte antigen; *eGFR*, estimated glomerular filtration rate; *ACEI*, angiotensin-converting enzyme inhibitor

### Dehydration admissions

In the first 12 months post-transplantation, 39/92 (42%) transplant recipients had at least 1 dehydration admission. There were 13 recipients with more than 1 dehydration admission. There were 26/55 (47.27%) dehydration admissions associated with poor intake, and 4 of these were recurrent poor intake admissions. January, which is mid-summer in our state, was the most common month of admission for poor intake (Fig. 2). For the 29 dehydration admissions associated with contributing illness, the specific contributing illnesses are listed in Table 2. Contributing illness dehydration admissions occurred throughout the calendar year (Fig. 2). Dehydration admissions accounted for 55/131 (41.98%) of all unplanned admissions, with poor intake admissions accounting for 19.85% of unplanned admissions (26/131).

**Table 2:** Types of contributing illness-associated dehydration (*N* = 29)

Febrile illness of unclear aetiology	2
Upper or lower respiratory tract infection	8
Vomiting illness	3
Diarrhoeal illness	10—potentially mycophenolate*—associated in 5
Vomiting and diarrhoea	3—potentially mycophenolate*—associated in 2
Pain of unclear aetiology	1
Cytomegalovirus disease	1
Shingles	1

Potential mycophenolate diarrhoea was recorded where the admission medical record noted this as a possibility, or where mycophenolate dosing or prescription during a diarrhoeal illness was changed to azathioprine, three times daily mycophenolate or enteric-coated mycophenolate

Poor intake admissions occurred more frequently in the early period after transplantation with a median admission time of 49.5 days after the transplant date (IQR 30–77). This compared with contributing illness admissions which distributed more evenly over the 12 months post-transplant (Fig. 2), with a mean admission time of 155.34 days (SD 100.56).

Median length of poor intake admissions was 2 days (IQR 2–3). Median percentage creatinine rise at the time of admission from the prior three creatinine tests was 31.5% (IQR 23–41). AKI by KDIGO definition with a ≥ 50% increase from baseline creatinine occurred in 3 (11.5%) poor intake admissions. Biopsies were undertaken in 9/26 (34.62%) poor intake admissions. No pathology was described in 7 of these, donor vessel change was reported in 1, and 1 biopsy was reported to demonstrate subtle tubular epithelial thinning and changes suggestive of acute tubular necrosis (ATN) with calcineurin inhibitor damage. HLA antibody testing and a renal tract ultrasound were respectively undertaken in 4/26 (15.38%) and 14/26 (53.85%) poor intake admissions.

For contributing illness dehydration admissions, median admission length was also 2 days (IQR 2–3). Median percentage creatinine rise at the time of admission was 52% (IQR 38–68). AKI occurred in 14 admissions (48.26%), with 10/14 at stage 1 and 4/14 at stage 2 AKI based on the KDIGO definition. No patients required dialysis for AKI. A biopsy was undertaken only for 1 patient with the finding of ATN. A renal tract ultrasound was undertaken in 5/29 patients and 1 patient had HLA antibody testing.

### Risk factors for dehydration admissions

Univariate models are presented in Table 3. Centre of transplant care did not associate with admissions. In adjusted models (Table 4), fluid intake > 100 ml/kg at discharge associated with all dehydration admissions. The teen age bracket at transplantation was associated with higher hazards of poor fluid intake dehydration admission, but this demonstrated wide confidence intervals. At the opposite end of the age spectrum, children under 4 years of age had a higher hazard of a dehydration admission associated with contributing illness but this was not significant in the adjusted model (removed as this rendered the model insignificant). Use of a gastrostomy or feeding tube at discharge associated with higher hazards of contributing illness dehydration, 80% of which were an illness with gastrointestinal losses. There was a moderate correlation (Pearson’s correlation coefficient = 0.52) identified between age under 4 years and enteric tube feeding. Given substantial missing data for native urine output volume pre-transplant, this variable was excluded from regression analyses. Where data was available however, median native output was significantly higher with poor intake dehydration admissions (*p* = 0.023). There was no significant difference in median native urine output for those with all category dehydration admission versus no admissions, or contributing illness dehydration admissions. For transplant recipients with native urine output data available, this did not correlate with target fluid intake at discharge.

**Table 3:** Univariable model for risk of all hospital admissions associated with dehydration

	All dehydration admissions, *N* = 55 HR (95% CI), *p* value	All poor intake dehydration admissions, *N* = 26 HR (95% CI), *p* value	All contributing illness dehydration admissions, *N* = 29 HR (95% CI), *p* value
Baseline recipient factors			
Gender			
Male	1.40 (0.72–2.73), 0.32	1.61 (0.52–4.92), 0.41	1.25 (0.56–2.79), 0.59
Primary kidney disease			
CAKUT	6.22 (0.97–40.05), 0.054	3.07 (0.44–20.72), 0.25	4.70 (0.72–30.48), 0.11
Cystic	Ref	Ref	No events
Nephronophthisis	1.28 (0.10–15.78), 0.85	No events	1.89 (0.15–23.53), 0.62
Glomerulonephritis	6.43 (0.93–44.63), 0.060	2.29 (0.19–27.33), 0.51	5.99 (0.71–50.47), 0.10
Steroid resistant or congenital nephrotic	6.34 (0.91–44.22), 0.062	2.60 (0.26–26.29), 0.42	5.50 (0.76–39.28), 0.091
Ischaemic	5.0 (0.71–36.83), 0.11	3.10 (0.39–24.30), 0.28	2.99 (0.35–25.78), 0.32
Other	1.35 (0.15–11.88), 0.79	0.67 (0.053–8.35), 0.75	Ref
Age at transplant			
< 4 years	2.12 (0.85–5.29) 0.11	3.64 (0.71–17.75), 0.12	**2.88 (1.22–6.78), 0.016**
4–12 years	Ref	Ref	1.60 (0.59–4.26), 0.35
> 12 years	1.457 (0.65–3.26), 0.36	**4.87 (1.19–19.86), 0.028**	Ref
Anuria pre-transplant	1.03 (0.55–1.94), 0.92	1.13 (0.41–3.15), 0.80	0.95 (0.36–2.53), 0.92
Transplant factors			
Past failed transplant	0.72 (0.31–1.65), 0.43	1.36 (0.43–4.25), 0.60	0.25 (0.036–1.70), 0.16
Transplant type			
Deceased donor	Ref	Ref	Ref
Live donor	1.41 (0.75–2.64), 0.28	1.75 (0.65–4.67), 0.27	1.18 (0.49–2.87), 0.71
No prior dialysis (pre-emptive live donor transplant)	0.63 (0.30–1.32), 0.22	0.76 (0.23–2.51), 0.66	0.52 (0.18–1.50), 0.22
ABO or HLA sensitised	0.25 (0.041–1.47), 0.13	0.50 (0.08–2.99), 0.45	No events
Transplantation during Australian summer (December–February)	1.30 (0.74–2.32), 0.36	1.36 (0.62–2.98), 0.44	1.25 (0.54–2.90), 0.61
Gastrostomy tube or feeding tube at discharge	1.40 (0.81–2.42), 0.22	0.62 (0.24–1.62), 0.33	**2.68 (1.32–5.45), 0.007**
Delayed graft function	1.20 (0.62–2.33), 0.59	0.76 (0.18–3.21), 0.71	1.63 (0.66–4.03), 0.29
Discharge target fluids > 100 ml/kg/day	**2.02 (1.23–3.31), 0.005**	1.80 (0.83–3.87), 0.14	**2.25 (1.12–4.53), 0.023**
Hyperfiltration at discharge (> 135 ml/min/1.73 m^2^)	1.12 (0.55–2.27), 0.75	1.17 (0.44–3.14), 0.76	1.08 (0.48–2.43), 0.86

Bolded values indicate* p < *0.05

*HR*, hazard ration; *CI*, confidence interval; *N*, number; *IQR*, interquartile range; *Ref*, reference; *ABO*, ABO blood group; *HLA*, human leukocyte antigen; *GFR*, glomerular filtration rate

**Table 4:** Multivariable model for risk of all hospital admissions associated with dehydration

	All dehydration admissions HR (95% CI), *p* value	All poor intake dehydration admissions HR (95% CI), *p* value		All contributing illness dehydration admissions HR (95% CI), *p* value	
Primary kidney disease		Age at transplant		Gastrostomy or feeding tube at discharge	**2.18 (1.08–4.41), 0.030**
CAKUT	56.68 (0.93–48.1), 0.059	< 4 years	3.64 (0.71–17.75), 0.12		
Cystic	Ref	4–12 years	Ref	Discharge target fluids > 100 ml/kg/day	1.62 (0.85–3.12), 0.15
Nephronophthisis	1.32 (0.09–19.09), 0.84	> 12 years	**4.87 (1.19–19.86), 0.028**		
Glomerulonephritis	**9.21 (1.11–76.57), 0.040**				
Steroid resistant or congenital nephrotic	5.65 (0.76–42.27), 0.092				
Ischaemic	4.32 (0.53–34.84), 0.17				
Other	1.70 (0.17–17.10), 0.65				
Discharge target fluids > 100 ml/kg/day	**2.04 (1.13–3.68), 0.018**				

Bolded values indicate* p < *0.05

*HR*, hazard ration; *CI*, confidence interval; *N*, number; *IQR*, interquartile range; *Ref*, reference; *ABO*, ABO blood group; *HLA*, human leukocyte antigen; *GFR*, glomerular filtration rate

## Discussion

In this cohort, dehydration admissions in the first 12 months following childhood kidney transplantation were common. Poor intake dehydration admissions accounted for almost half of dehydration admissions and approximately 1 in 5 of all unplanned admissions. AKI occurred in almost half of dehydration admissions associated with contributing illness.

Although poor intake admissions were usually brief, a kidney biopsy was undertaken in around one third, and for 1 in 5, a further poor intake admission occurred. The median time of these poor intake admissions at approximately 2 months post-transplantation, and frequency of biopsies, could suggest that the main objective of the admission was to exclude alternative diagnoses at a time period where rejection risk is considered high. More complex logistics of arranging biopsies in children, where fasting, sedation, or general anaesthetic is often required, may also have meant that biopsies were arranged, even when creatinine had already improved or prior to full fluid challenge. Early poor intake admissions could also reflect poor early post-transplant renal concentrating capacity. This is speculative as no routine assessment of renal concentrating capacity was undertaken in the post-transplant period in this cohort.

The association of high discharge fluid intake targets with all dehydration admissions is plausible. The risk in this cohort appears driven predominately by a higher number of contributing illness dehydration admissions, although this variable was not significant in the adjusted analysis. If high fluid targets at discharge represent true fluid requirement, then this association may represent poor renal transplant concentrating capacity and/or high urine output from native kidneys. Such patients may have vulnerabilities in achieving higher fluid targets, especially in illness impacting intake, or where there are additional losses. The high median native urine output for those with subsequent poor intake dehydration admissions again suggests that this factor could be a key contributor to dehydration risk; however, missing data limits further direct interpretation in our cohort. In our cohort, native nephrectomies were undertaken post-transplant in 4 of the 39 recipients with a dehydration admission, with no subsequent dehydration admissions up to 12 months post-transplant, suggesting that native urine output is important. Other authors have discussed high native urine output as a risk for graft hypoperfusion and requirement for large fluid intake which has influenced decision-making about native nephrectomies [3].

Contrary to our hypothesis, the lack of significance of fluid intake targets in the univariate model for poor intake admissions could suggest that when well, most patients are able to meet their fluid target. This also appears to include children with anuria pre-transplant, as we could not find any evidence that this associates with poor fluid intake dehydration admissions. An alternative explanation is that the fluid target is higher than what is actually required in the absence of illness. As discussed by Wyatt et al., the process of prescribing fluid volumes post-transplant has inherent inaccuracies, as when we prescribe increased volumes, we may drive urine output [12]. Our own practice of prescribing fluid targets at discharge, as described in our methods, may be inaccurate for the same reason. Also, there were many factors that we were unable to assess in this retrospective study that would be important to help understand how fluid targets are derived. We agree with the conclusions of Wyatt et al. that further studies of optimal fluid intake prescriptions are warranted in paediatric kidney transplantation.

Whilst season of the date of transplantation did not associate with dehydration admissions, the maximum per-month admissions for poor intake dehydration occurred in our mid-summer, January. Contributing illness dehydration admissions occurred more regularly throughout the year. Average maximum daily temperature in January in Melbourne, the capital city of Victoria, for the same 11-year period was 27.62 °C (81.72 °F). This compared with an average annual maximum daily temperature of 20.66 °C (69.19 °F) [17]. It is possible that the most frequent month of transplantation being November may also be the reason for the apparent mid-summer admission phenomenon. The identified association between age greater than 12 years and poor intake dehydration admissions could suggest increased social or other adherence challenges associated with developing independence. This may also be a particular problem in January in Victoria, Australia, given that most of this month is the long school holiday break.

Risk of admissions with contributing illness dehydration will be theoretically linked with risk factors for those contributing illnesses. The severity of the contributing illness may also be a key reason for admission, rather than the graft dysfunction. The main indication for admission in these patients was not able to be assessed in this study. A detailed analysis including time-varying immunosuppression would also be required to fully assess the risk in this population which was beyond the scope of this research. The association of gastrostomy or feeding tube use at transplant discharge with a higher risk of contributing illness dehydration admission was unexpected. It is possible that there are confounding factors that contribute to this risk, including younger age at transplant. The age categorisation used in this study was based on a hypothesis regarding parental capacity to supervise fluid intake. Results however suggest that this categorisation could also reflect risk of concurrent illness and especially infections with associated fluid loss or poor intake. It is possible that the use of a gastrostomy tube could also identify patients with feed intolerance that is exacerbated with contributing illness. Contrary to our hypothesis, the presence of a gastrostomy tube does not appear to be a protective factor in this setting.

Contributing illness dehydration admissions with AKI were frequent in this cohort. Further studies are required to understand whether this may have any impact on graft function over time, especially in the setting of recurrent episodes.

There are a number of study limitations that need to be considered as part of interpreting our results. The cohort size is small, and thus, the assessment of risk for some specific variable categories is limited. Given the retrospective nature of this analysis, we were also limited in the breadth and depth of the variables that could be assessed, due to limited detailed recording in the medical records, such as for native urine output. All dehydration admissions meeting our outcome criteria into the tertiary hospitals were accounted for in this analysis. We also searched all available records to help ensure that any secondary (regional) hospital admissions were also recorded. There were none identified. This was expected based on the management practice in our state; however, we cannot guarantee that there was no missing dehydration admission data. Restriction of our cohort to two transplant centres within the same state may limit the generalisability of our findings. For example, it is possible that in centres undertaking early protocol biopsies, admissions for poor intake may be less common. However, in presenting a detailed description of the cohort and our fluid and immunologic management strategies, comparisons can potentially be made for other transplant centres. It should be noted that our use of the term dehydration reflects the pragmatic approach to management of this clinical presentation. Our definition solely was based on the improvement of creatinine with rehydration, and no other specific diagnostic markers of dehydration were evaluated. We also cannot be sure that there were no other reasons for graft dysfunction especially in the contributing illness dehydration group. This includes calcineurin inhibitor nephrotoxicity, which can occur with target levels below the definition we have used [18]. For the single kidney transplant state, calcineurin inhibitor nephrotoxicity–associated reduced glomerular perfusion [19] could be an important mechanism underlying creatinine rise in the setting of mild dehydration.

## Conclusion

This is the first published report to our knowledge describing frequency and risk factors for dehydration admissions following kidney transplantation, in either children or adults. Anecdotally, prior to this analysis, our experience had been that poor intake admissions were frequent. This has been confirmed now with a formal retrospective review. The key risk factor identified for all dehydration admissions was higher target fluid intake at discharge. A higher number of poor intake admissions occurred in the teen age group and in mid-summer. Contributing illness dehydration admissions were predicted by the use of an enteric feeding tube, which may be a marker of contributing illness risk associated with younger age. Further studies are required to understand whether the current practice of prescribing fluid intake targets and the hydration advice given to patients, especially in the setting of illness or hot weather, can be improved. This may be worthwhile to reduce admissions and everyday management challenges for transplant recipients and their families.

### Supplementary Information

Below is the link to the electronic supplementary material.

Graphical abstract (PPTX 60 KB)

## Data Availability

Data are available upon request to the corresponding author.
