# Supplementary material for: Hospital admissions associated with dehydration in childhood kidney transplantation
Source: Pediatr Nephrol. 2023 Aug 9;39(2):547–57. doi: 10.1007/s00467-023-06095-6 (PMC10728223; doi:10.1007/s00467-023-06095-6)
Supplement: Supplementary file 1 — Graphical abstract (PPTX 60 KB) [file 467_2023_6095_MOESM1_ESM.pptx]

## Slide 1
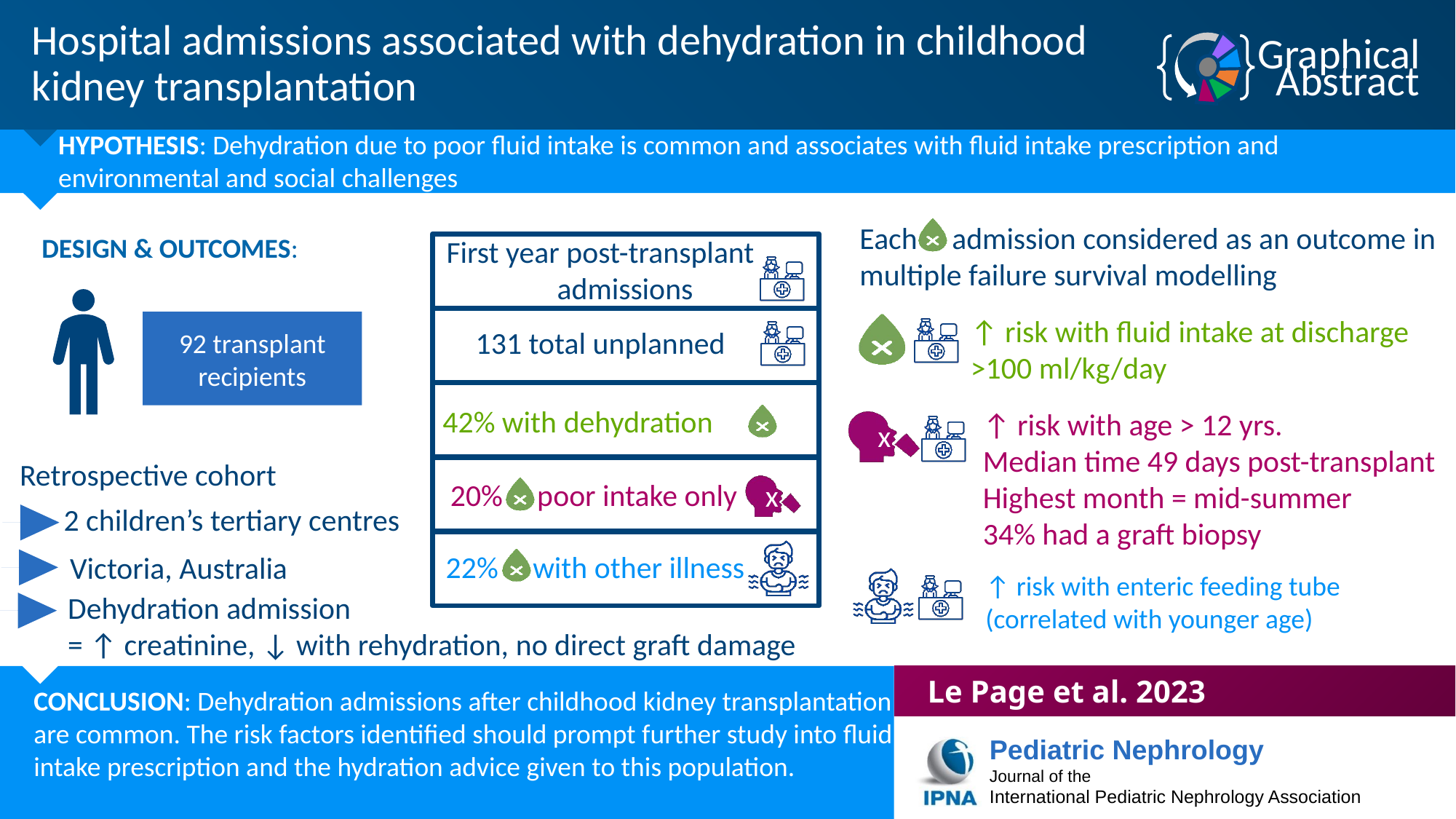

Hospital admissions associated with dehydration in childhood kidney transplantation
HYPOTHESIS: Dehydration due to poor fluid intake is common and associates with fluid intake prescription and environmental and social challenges
Each admission considered as an outcome in multiple failure survival modelling
DESIGN & OUTCOMES:
First year post-transplant
 admissions
↑ risk with fluid intake at discharge
>100 ml/kg/day
92 transplant recipients
131 total unplanned
42% with dehydration
↑ risk with age > 12 yrs.
Median time 49 days post-transplant
Highest month = mid-summer
34% had a graft biopsy
x
Retrospective cohort
20% poor intake only
x
2 children’s tertiary centres
22% with other illness
Victoria, Australia
↑ risk with enteric feeding tube (correlated with younger age)
 Dehydration admission
 = ↑ creatinine, ↓ with rehydration, no direct graft damage
Le Page et al. 2023
CONCLUSION: Dehydration admissions after childhood kidney transplantation are common. The risk factors identified should prompt further study into fluid intake prescription and the hydration advice given to this population.
